# A Type Ia Crustin from the Pacific White Shrimp *Litopenaeus vannamei* Exhibits Antimicrobial and Chemotactic Activities

**DOI:** 10.3390/biom15071015

**Published:** 2025-07-14

**Authors:** Xiuyan Gao, Yuan Liu, Xiaoyang Huang, Zhanyuan Yang, Mingzhe Sun, Fuhua Li

**Affiliations:** 1 School of Marine Science and Engineering, Qingdao Agricultural University, Qingdao 266109, China; xygao@qdio.ac.cn; 2 Key Laboratory of Breeding Biotechnology and Sustainable Aquaculture (CAS), Institute of Oceanology, Chinese Academy of Sciences, Qingdao 266071, China; 18169272524@163.com (X.H.); yzyqdu@163.com (Z.Y.); mzhsun@qdio.ac.cn (M.S.); fhli@qdio.ac.cn (F.L.); 3 Laboratory for Marine Biology and Biotechnology, Qingdao Marine Science and Technology Center, Qingdao 266071, China

**Keywords:** *Litopenaeus vannamei*, crustin, antimicrobial peptides, chemotaxis, innate immunity

## Abstract

Crustins are a family of cysteine-rich antimicrobial peptides (AMPs), predominantly found in crustaceans, and play important roles in innate immunity. However, among the many reported crustins, few studies have explored their immunomodulatory functions. In this study, we investigated the immune function of a type I crustin (LvCrustinIa-2) in *Litopenaeus vannamei*, with particular emphasis on comparing the roles of its different domains. LvCrustinIa-2 possesses cationic patchy surface and amphipathic structure, and its expression was significantly induced in hemocytes after pathogen challenge. Both the recombinant LvCrustinIa-2 (rLvCrustinIa-2) and its whey acidic protein (WAP) domain (rLvCrustinIa-2-WAP) exhibited significant inhibitory activities against the tested Gram-positive bacteria. They also showed binding affinity not only for Gram-positive bacteria but also for Gram-negative bacteria. Furthermore, rLvCrustinIa-2 induced membrane leakage and structure damage in the target bacteria. Notably, chemotaxis assays revealed that rLvCrustinIa-2 and the synthetic cysteine-rich region (LvCrustinIa-2-CR) significantly enhanced the chemotactic activity of shrimp hemocytes in vitro. Knockdown of *LvCrustinIa-2* triggered significant transcriptional activation of genes involved in calcium transport, inflammation, redox regulation, and NF-κB pathway. Taken together, these findings elucidate the distinct roles of the cysteine-rich region and WAP domain in type Ia crustin and provide the first evidence of a crustacean AMP with chemotactic and immunomodulatory activities.

## 1. Introduction

Antimicrobial peptides (AMPs), distributed in almost all organisms, are key effector molecules in the innate immune response [1,2]. They are a class of small peptides that exhibit a broad-spectrum activity against bacteria, fungi, viruses and parasites [3,4]. Most AMPs are amphiphilic and cationic in nature, performing their antimicrobial effects mainly through interacting with negatively charged microbial membranes. This interaction could disrupt membrane integrity and lead to cell membrane lysis or cell content release [5]. Given their rapid microbicidal action and low propensity for resistance development, AMPs have emerged as promising candidates for combating the growing threat of multidrug-resistant pathogens [6].

In addition to antimicrobial activity, more studies have indicated that AMPs exhibit diverse immunomodulatory functions, such as chemotaxis stimulation, induction of pro- and anti-inflammatory cytokines, endotoxin neutralization, and activation and differentiation of immune cell lines [7,8,9,10]. For instance, the human cathelicidin antimicrobial peptide LL-37 could promote inflammatory cell recruitment by stimulating leukocyte chemotaxis [11] and selectively suppress lipopolysaccharide (LPS), and LPS/interferon-γ (IFN-γ)-induced tumor necrosis factor-α (TNF-α) and nitric oxide (NO) production in macrophages [12]. Human β-defensins share structural similarities with selected chemokines and have chemotactic activity by attracting dendritic cells, monocytes and T-lymphocytes to the site of infection [13,14]. The chemotactic role of β-defensins has also been reported in teleost fish, such as the gilthead seabream (*Sparus aurata*) [15], cyprinid fish (*Megalobrama amblycephala*) [16], and flounder (*Paralichthys olivaceus*) [17]. However, studies of immunomodulatory activity among AMPs in invertebrates remain limited, with only a few mollusk peptides (defensins and macins) demonstrating chemotactic activity toward hemocytes [18,19,20].

In crustaceans, multiple AMP families, such as crustins, anti-lipopolysaccharide factors, penaeidins, and stylicins, have been identified and characterized [21,22,23]. Crustins are cationic cysteine-rich AMPs characterized by the C-terminal whey acidic protein (WAP) domain [24]. The WAP domain, essential for the biological activity of crustins, contains eight conserved cysteine residues forming a four-disulfide core (4DSC) [25,26]. Crustins can be classified into diverse types based on the variable N-terminal region [25,27]. Type I crustins are characterized by the presence of a cysteine-rich region located between the signal peptide and the WAP domain, and can be further classified into three subtypes, i.e., Ia, Ib, and Ic. Type II crustins consist of a glycine-rich region and a cysteine-rich region, while type III crustins have a short N-terminal region enriched in proline/arginine residues [25]. It has been reported that all these crustins possess antibacterial, anti-fungal, and proteinase inhibitory activities [22,28,29,30]. However, the potential roles of crustins in other immune processes remain unexplored.

The Pacific white shrimp *Litopenaeus vannamei* has become the dominant crustacean species in aquaculture worldwide. However, shrimp diseases, particularly induced by *Vibrio parahaemolyticus* and white spot syndrome virus (WSSV), have posed a severe threat to the development of the shrimp farming industry [31,32]. Studies on AMPs will be useful in understanding the immune defense mechanisms of shrimp and gaining new insights into diseases control in shrimp aquaculture. In our previous study, a type Ia crustin gene, named *LvCrustinIa-2*, was identified from *Litopenaeus vannamei* [33]. It was mainly detected in hemocytes and intestines, and the knockdown of *LvCrustinIa-2* could impair the balance of intestinal microbiota. To further explore the immune function of LvCrustinIa-2, we analyzed its structural features, examined its temporal expression pattern, and compared the biological activities of its different domains. The antimicrobial activity, microbial binding, and particularly the chemotactic activity were evaluated using synthetic cysteine-rich peptide and recombinant LvCrustinIa-2 and its WAP domain. Moreover, by double-stranded RNA (dsRNA)-mediated RNA interference (RNAi), the role of *LvCrustinIa-2* in the regulation of calcium transport, inflammation, and immune signaling was investigated.

## 2. Materials and Methods

### 2.1. Experimental Shrimp and Tissue Collection

Healthy shrimp *L. vannamei* (5.21 ± 0.56 g) were purchased from the local farm in Rizhao, Shandong Province, China, and cultured in our laboratory for two weeks with aerated seawater at 25 ± 1 °C and fed three times daily.

For the immune challenge experiment, shrimp were randomly allocated into control group, *V. parahaemolyticus*-challenged group, and WSSV-challenged group, and each group contained 80 individuals. *V. parahaemolyticus* and WSSV were diluted with PBS to concentrations of 5 × 10^4^ colony forming unit (cfu)/tail and 1 × 10^3^ copies/tail, respectively. Using a microinjector, a 10 µL dose of the diluted pathogen was injected into the muscle of the third or fourth ventral segment of each shrimp. Shrimp injected with PBS served as the control group. The injected shrimps were returned to the water tanks and sampled at the time points of 0, 6, 12, 24, and 48 h post-injection.

Hemolymph was extracted from the ventral sinus located at the first abdominal segment of shrimp using a sterile syringe with an equal volume of precooled anticoagulant solution (115 mmol L^−1^ glucose, 27 mmol L^−1^ sodium citrate, 336 mmol L^−1^ NaCl, 9 mmol L^−1^ EDTA·Na2·2H_2_O, pH 7.4) [34]. The hemolymph was immediately centrifuged at 800× *g*, 4 °C for 8 min to separate the hemocytes. The hemocytes were collected at the bottom of the tube and frozen in liquid nitrogen.

### 2.2. Total RNA Extraction and cDNA Cloning

Total RNA was extracted using RNAiso Plus reagent (TakaRa, Dalian, China) according to the manufacturer’s instructions. The cDNA template was synthesized using PrimeScript™ II 1st cDNA Synthesis Kit (TakaRa, Dalian, China).

Based on the unigene sequence, a pair of primers, LvCrustinIa-2F and LvCrustinIa-2R (Table 1), were designed to amplify the open reading frame (ORF) of *LvCrustinIa-2*. The polymerase chain reaction (PCR) mixture (25 µL total volume) contained 12.5 µL of 2 × Accurate Taq Master Mix (Accurate Biotechnology, Changsha, China), 9.5 µL nuclease-free water, 1 µL of each primer (10 µmol L^−1^), and 1 µL of cDNA template (50 ng). The amplification procedure was initiated with 5 min at 95 °C, followed by 35 cycles of 30 s at 95 °C, 30 s at 58 °C, and 30 s at 72 °C, with a final extension for 10 min at 72 °C. After amplification, the PCR products were purified using Mini BEST DNA fragment purification kit (TakaRa, Dalian, China). The purified fragments were then ligated into the pMD19-T vector (TakaRa, Dalian, China) and transformed into *Escherichia coli* Trans5α competent cells (TransGen Biotech, Beijing, China). The positive colonies were sent to Sangon Biotech Company (Shanghai, China) for sequencing.

### 2.3. Bioinformatics and Structural Characterization

The nucleotide sequence and deduced amino acid sequence of LvCrustinIa-2 were analyzed by using the online BLAST algorithm (http://blast.ncbi.nlm.nih.gov/Blast.cgi (accessed on October 2024)). The protein domain was predicted by SMART (http://smart.embl.de (accessed on 10 October 2024)). The structure model of LvCrustinIa-2 was constructed using AlphaFold3 server (https://alphafoldserver.com (accessed on 15 April 2025)), and structural images were generated using Pymol Molecular Graphics System 2.5.0. (https://pymol.org (accessed on 15 April 2025)).

### 2.4. Real-Time Quantitative PCR Analysis

The expression of *LvCrustinIa-2* in hemocytes of shrimp challenged with *V. parahaemolyticus* and WSSV was determined by specific primers qLvCrustinIa-2F and qLvCrustinIa-2R using real-time quantitative PCR. The cDNA was diluted 40-fold with deionized water. The qPCR was conducted in a 10 µL reaction mixture comprising 3.33 µL of 2 × SYBR Premix ExTaq (TakaRa, Dalian, China), 0.13 µL of 50 × ROX Reference Dye, 2.28 µL of sterile distilled H_2_O, 0.13 µL of each primer (10 µmol L^−1^), and 4 µL of diluted cDNA. The qPCR amplification procedure was as follows: 95 °C for 35 s, followed by 32 cycles of 95 °C for 10 s and 60 °C for 30 s, 72 °C for 1 min, and finally 72 °C for 2 min. Each sample was analyzed in triplicate. The relative expression level of *LvCrustinIa-2* was determined using the 2^−ΔΔCT^ method, with 18S rRNA as the internal reference. Data were analyzed by one-way ANOVA using SPSS 25.0 software, with *p* < 0.05 indicating statistical significance.

### 2.5. Expression and Purification of Recombinant Proteins

The sequence encoding the mature peptide and the WAP domain were amplified by primers rLvCrustinIa-2F/R and rLvCrustinIa-2-WAP-F/R (Table 1). The expression vector pET28a was linearized using restriction enzymes *Bam* HI and *Hind* III (TakaRa, Dalian, China). Subsequently, the purified PCR fragment was ligated into the digested vector with the In-Fusion HD Cloning Kit (TakaRa, Dalian, China). The successfully sequenced recombinant expression plasmids pET28a-LvCrustinIa-2, pET28a-LvCrustinIa-2-WAP, and the empty vector pET-32a were transformed into *E. coli* BL21 (DE3) competent cells (Transgen, Beijing, China). The soluble recombinant proteins LvCrustinIa-2, LvCrustinIa-2-WAP and thioredoxin (rTrx) were induced by the addition of isopropyl-β-D-thiogalactoside (IPTG) at a final concentration of 0.5 mmol L^−1^ for 6 h at 28 °C. Cells were harvested by centrifugation, resuspended in buffer I (50 mM sodium phosphate, 300 mM NaCl, pH 7.0), and sonicated at 4 °C for 30 min in a combination of 2 s sonication and 2 s interval under 180 W power. To obtain recombinant proteins, rLvCrustinIa-2, rLvCrustinIa-2-WAP, and rTrx were purified under native conditions using TALON Metal affinity resin (Clontech, Mountain View, CA, USA) and dialyzed in 50 mmol L^−1^ Tris-HCl (pH 8.0) for 18 h. The purified proteins were collected using Amicon Ultra-15 10K MWCO devices (Millipore, Cork, Ireland). The proteins were determined using BCA Protein Assay Kit (Beyotime, Shanghai, China).

### 2.6. Peptide Design and Synthesis

The cysteine-rich region of LvCrustinIa-2, designated as LvCrustinIa-2-CR, was chemically synthesized in primary structure using a peptide synthesizer from Sangon Biotech (Shanghai, China). To neutralize the terminal charges and enhance the stability of peptide, the amino acid residue of LvCrustinIa-2-CR, Ac-CRRWCRTPEQQAYCCETVFEPEAPVGT-NH_2_, was acetylated in the N-terminus and amidated in the C-terminus. The synthesized peptide was analyzed by mass spectrometry. The observed molecular weight of the peptide was 3.20 kDa, which was the same as the theoretical molecular mass. The purity of the peptide was above 95% as verified by high-performance liquid chromatography (HPLC). For HPLC analysis, 1 mg mL^−1^ peptide in 20% acetonitrile (ACN) and 80% H_2_O was injected on SHIMAZU Shim-pack GIST C18 column (4.6 × 250 mm, 5 μm) under a gradient of 25–45% B (A = 0.1% trifluoroacetic acid in 100% water; B = 0.1% trifluoroacetic acid in 100% acetonitrile) over 20 min at a flow rate of 1.0 mL min mL^−1^, and the retention time was 11.23 min.

### 2.7. Minimal Inhibitory Concentration (MIC) Assay

Antimicrobial activity was measured against four Gram-negative bacteria *V. parahaemolyticus* Pa6, *V. alginolyticus* L59, *Pseudomonas aeruginosa* P25, and *Edwardsiella tarda* E3, and three Gram-positive bacteria *Staphylococcus aureus* S7, *S. delphini* Sd2, and *Micrococcus luteus* M2, and one fungus *Pichia pastoris* GS115 in our lab, using a liquid phase assay modified from that of Rathinakumar et al. [35]. MICs were assessed using serial two-fold microdilutions according to Clinical and Laboratory Standards Institute (CLSI) protocol [36]. rLvCrustinIa-2, rLvCrustinIa-2-WAP, and synthetic LvCrustinIa-2-CR were in 1/2-fold serial dilution with Tris-HCl (50 mmol L^−1^, pH 8.0). *V. parahaemolyticus* Pa6, *V. alginolyticus* L59, *P. aeruginosa* P25, and *E. tarda* E3 were cultured in tryptic soy broth (TSB) + 2% NaCl medium at 28 °C, *S. aureus* S7, *S. delphini* Sd2, and *M. luteus* M2 were cultured in LB medium at 37 °C, and *P. pastoris* GS115 was cultured in yeast extract peptone dextrose (YPD) medium at 28 °C. All microorganisms were cultured to logarithmic phase (absorbance of 0.6), and diluted to 1 × 10^3^ cfu mL^−1^ with filtered sterilized 50 mmol L^−1^ Tris-HCl (pH 8.0). In 96-well plates, 50 μL of density-gradient recombinant proteins and 50 μL of bacterial suspension were added to each well. The wells with 50 µL of Tris-HCl (50 mmol L^−1^, pH 8.0) and 50 µL of rTrx diluted with Tris-HCl (50 mmol L^−1^, pH 8.0) were used as the blank group and the negative control, respectively. The 96-well plates were incubated at corresponding temperatures for up to 2 h. After incubation, 150 µL of corresponding growth medium was added, and the mixtures were allowed to recover overnight. Microbial growth was monitored by measuring absorbance at 560 nm (Gram-negative bacteria and fungus) or 600 nm (Gram-positive bacteria) using a TECAN Infinite M200PRO microplate reader (Salzburg, Austria). Experiments were performed in triplicate. MIC was determined as the lowest concentration of protein at which no detectable bacterial growth was observed.

### 2.8. Microbial Binding Assay

The microbial binding activity of recombinant proteins and synthetic peptide was assayed against two Gram-negative bacteria, *V. parahaemolyticus* Pa6 and *V. alginolyticus* L59, two Gram-positive bacteria, *S. aureus* S7 and *M. luteus* M2, and one fungus, *P. pastoris* GS115, according to a previously described method [37]. The microorganisms were cultured in the corresponding medium to logarithmic growth phase, fixted with 4% formaldehyde, and washed three times with Tris-HCl buffer (50 mmol L^−1^, pH 8.0). The microorganism and protein (final concentration 1 mg mL^−1^) were mixed in equal volumes, the mixture was centrifuged at 4000 rpm, 4 °C for 5 min, and the supernatant was collected. The bacterial pellets were washed thrice with 1 mL of PBS, and the wash fractions were collected. Subsequently, the bacteria were resuspended in PBS. The rTrx protein served as a negative control. The washed pellets, supernatant, and eluted fractions were analyzed via 15% SDS-PAGE.

### 2.9. Electron Microscopy

*V. parahaemolyticus* and *S. aureus* were cultivated in corresponding media to logarithmic phase, and diluted to 1 × 10^6^ cfu mL^−1^ with PBS. The bacterial suspension was mixed with rLvCrustinIa-2 (1.6 mg·mL^−1^) at 28 °C for 2 h. The bacteria-mixed PBS was used as the control group. After incubation, the microorganisms were fixed with 5% glutaraldehyde in PBS (pH 7.4) for 2 h, followed by dehydration in a series of increasing concentrations of ethanol (30%, 50%, 70%, 80%, 90%, and 100%) at 4 °C for 10 min each. The samples were treated with isoamyl acetate for 10 min, critical point-dried (Hitachi-HCP, Hitachi, Tokyo, Japan), sputter-coated with platinum (MC1000, Hitachi, Tokyo, Japan), and observed under a scanning electron microscope (S-3400N, Hitachi, Tokyo, Japan) [38].

### 2.10. In Vitro Chemotaxis Assay

The chemotactic activity of recombinant proteins and synthetic peptide towards shrimp hemocytes was determined using Transwell permeable supports (Corning, New York, NY, USA) as described previously [39], with minor modifications. Briefly, hemolymph was extracted from the ventral sinus of shrimp with a syringe containing twice the volume of ice-cold anticoagulant solution. After centrifugation, hemocytes were resuspended in Insect-XPRESS medium (Lonza, Walkersville, MD, USA) at approximately 1.0 × 10^6^ cells mL^−1^. The chemotatic assay was performed in 24-well Transwell plates with 8 µm pore size. The lower chamber was filled with 600 μL of rLvCrustinIa-2, rLvCrustinIa-2-WAP, or synthetic LvCrustinIa-2-CR in Insect-XPRESS™ medium with the final concentration of 0.15, 0.15, and 0.17 mg mL^−1^, respectively. And the upper chamber received 100 μL of hemocytes suspension. For the controls, 600 μL of rTrx or Tris-HCl was added to the lower chamber, respectively. After 4 h incubation at 28 °C with 5% CO_2_, non-migrated hemocytes were removed from the upper face of the membrane. After fixation in paraformaldehyde, the membrane was stained with 1% crystal violet staining solution (Solarbio, Beijing, China). The number of transmigrated hemocytes was counted in at least four fields with a light microscope (Olympus, Tokyo, Japan) at a magnification of 200×. Each assay was repeated independently three times. The chemotactic activity was defined as chemotactic index (CI), calculated as CI = number of migrated cells in experimental group/number of migrated cells in rTrx control group. A CI value greater than or equal to 2 would represent a positive chemotactic response.

### 2.11. RNA Interference Assay and Expression Changes in Immune Genes in the LvCrustinIa-2-Knockdown Shrimp

The dsRNA targeting *LvCrustinIa-2* was designed and assessed using E-RNAi version 3.0 (https://e-rnai.dkfz.de/signaling/e-rnai3/, accessed on 15 October 2024). A 358 bp fragment of *LvCrustinIa-2* was amplified from template plasmid pMD19-T-LvCrustinIa-2 using primers dsLvCrustinIa-2 F/R containing T7 promoter sequences (Table 1). The amplified product was analyzed by electrophoretic analysis and purified using the StablePure PCR DNA Purification Kit (Accurate Biology, Changsha, China). The purified DNA was then used as the template for dsRNA synthesis with the T7 High Yield Transcription Kit (Thermo Fisher Scientific, Waltham, MA, USA). The synthesized dsRNA was subsequently purified by phenol–chloroform extraction. The dsRNA concentration was determined using a NanoDrop 2000 spectrophotometer (Thermo Fisher Scientific, Wilmington, DE, USA), and its quality was confirmed by 1% agarose gel electrophoresis. Synthesized dsEGFP served as the negative control.

For RNA interference (RNAi) experiments, shrimp were allocated into six groups (15 shrimp per group), with three experimental groups receiving dsRNA-LvCrustinIa-2 injections and three control groups receiving dsEGFP (2.5 µg/g body weight). Each group contained three biological replicates (5 shrimp per replicate). Hemocytes were collected 48 h post-injection, and RNAi efficiency was determined using primers qLvCrustinIb-1-F/R through quantitative analysis.

Using qRT-PCR, the expression of several immune-related genes was analyzed in hemocytes of *LvCrustinIa-2*-knockdown shrimp. The immune-related genes included three calcium transport-related genes *LvNCX* (Na^+^/Ca^2+^ exchanger, XM_027355456.1), *LvPMCA-2* (plasma membrane calcium-transporting ATPase 2-like, XM_027379317.1) and *LvSERCA* (sarco/endoplasmic reticulum Ca^2+^-ATPase, JN986572.1), six inflammation-associated genes *LvLITAF* (lipopolysaccharide induced TNF-α factor, JN180640.1), *LvIL-16* (interleukin-16-like protein, KY052164.1), *LvNFIL3* (nuclear factor interleukin-3-regulated protein-like, XM_070134988.1), *LvTRAF6* (tumor necrosis factor receptor-associated factor 6, HM581680.1), *LvTNFSF* (JN180639.1) and *LvTNFRSF* (JN180641.1), six NF-κB pathway genes *LvToll1* (DQ923424.1), *LvToll2* (JN180637.1), *LvToll3* (JN180638.1), *LvDorsal* (dorsal, FJ998202.1), *LvCactus* (cactus protein, JX014314.1) and *LvRelish* (relish, EF432734.1), three JAK/STAT pathway genes *LvDOME* (domeless, KC346866.1), *LvJAK* (Janus kinase, KP310054.2) and *LvSTAT* (HQ228176), one MAPK pathway gene p38 (mitogen-activated protein kinase p38b-like, XM_027367739.1), and six redox-related genes included *LvNOS* (nitric oxide synthase, GQ429217.1), *LvNOX* (NADPH oxidase 5-like, XM_027352105.1), *LvGST* (glutathione S-transferase, AY573381.2), *LvDOUX* (dual oxidase 2-like, XM_027360938.1), *LvGPx* (glutathione peroxidase, AY973252.2) and *LvSOD* (cytosolic MnSOD, DQ005531.1).

## 3. Results

### 3.1. Characterization of LvCrustinIa-2

**Figure 1:**
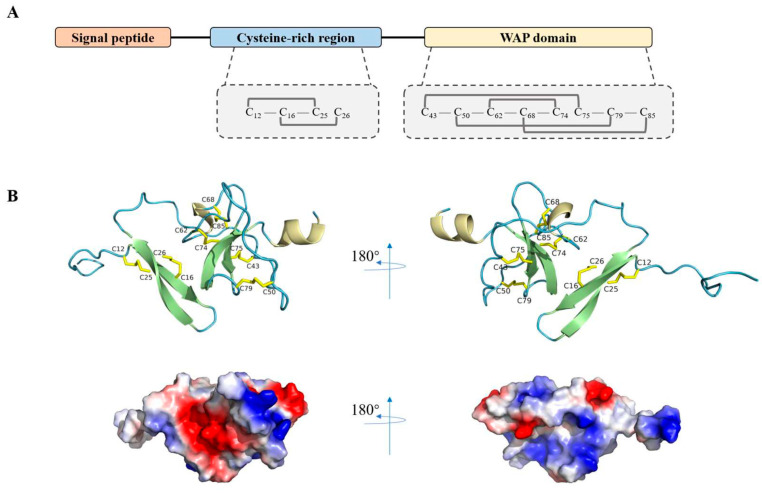
The analysis of the putative structure of LvCrustinIa-2. (**A**) Schematic illustration of domain structure of LvCrustinIa-2. The conserved cysteine residues in cysteine-rich region and WAP domain are denoted by dashed boxes. (**B**) The predicted spatial structure and net charge distribution of LvCrustinIa-2. β-sheets and α-helices are represented by broad arrows and spiral regions. The disulfide bonds are shown in yellow. The positive-charge regions are in blue, negative-charge regions are in red, and hydrophobic amino acids are in white.

*LvCrustinIa-2* (GenBank accession no. MT375558.1) contained a 345 bp open reading frame encoding 114 deduced amino acid polypeptide (Figure S1). The putative peptide contained an N-terminal signal peptide, a cysteine-rich region containing four conserved cysteine residues, and a C-terminal WAP domain with eight conserved cysteine residues (Figure 1A). The predicted spatial structure of putative LvCrustinIa-2 comprised an extended N-terminal coil followed by two-stranded antiparallel β-strands, a short 3_10_ helix, additional two β-strands, and a C-terminal α-helix (Figure 1B). Within the cysteine-rich region, two β-strands were proposed to be stabilized by two disulfide bonds (Cys12–Cys25 and Cys16–Cys26). The WAP domain primarily featured a typical ‘four-disulfide core’ structure, predicted to be formed by four disulfide bonds (Cys43–Cys75, Cys50–Cys79, Cys62–Cys74, and Cys68–Cys85). Notably, some adjacent cationic amino acids (highlighted in blue) could form a cationic cluster and be exposed on the surface of LvCrustinIa-2. The opposite side of this cluster was a hydrophobic surface composed of numerous hydrophobic amino acids (highlighted in white).

### 3.2. Expression Pattern Analysis of LvCrustinIa-2 After Pathogen Challenge

**Figure 2:**
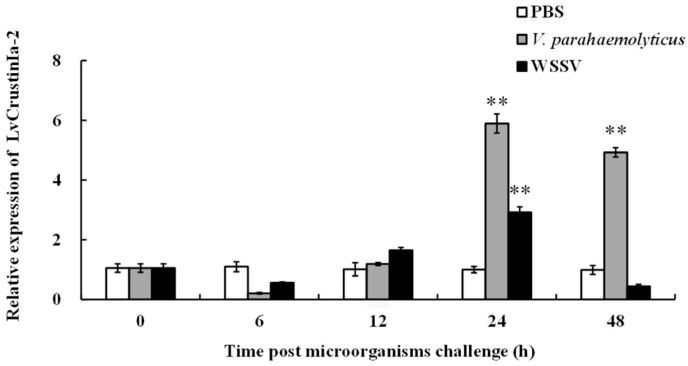
Relative expression of *LvCrustinIa-2* post challenge with *V. parahaemolyticus* (gray) and WSSV (black). Data are shown as mean ± S.D. (n = 3). Asterisks denote significant differences between the experiment and control groups at the same sampling point (** *p* < 0.01).

The temporal expression pattern of *LvCrustinIa-2* in hemocytes was investigated after challenge with *V. parahaemolyticus* and WSSV (Figure 2). During the first 6 h post-injection, *LvCrustinIa-2* expression decreased slightly compared to the control group. As time progressed, its expression was upregulated and reached the peak at 24 h post-injection, which was 5.89-fold compared to control (** *p* < 0.01) in the *V. parahaemolyticus*-challenged group and 2.92-fold compared to control (** *p* < 0.01) in the WSSV-challenged group. At 48 h post-injection, *LvCrustinIa-2* remained highly expressed in *V. parahaemolyticus*-challenged group (4.93-fold compared to control, ** *p* < 0.01), whereas it declined below the control level in the WSSV-challenged group.

### 3.3. Expression and Purification of the Recombinant LvCrustinIa-2 and LvCrustinIa-2-WAP

**Figure 3:**
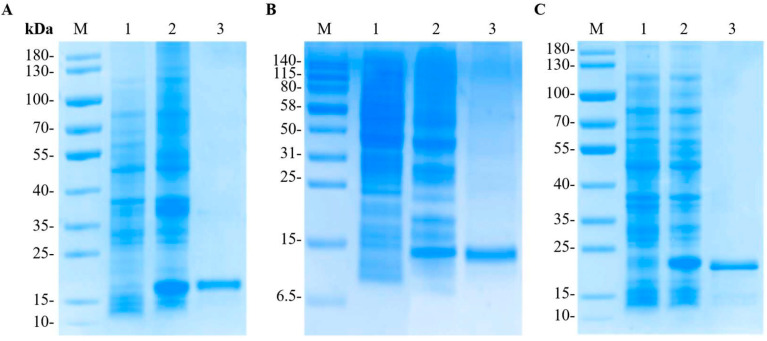
Recombinant expression and purification of rLvCrustinIa-2 (**A**), rLvCrustinIa-2-WAP (**B**), and rTrx (**C**). M, protein molecular standard (kDa); lane 1, total protein of *E. coli* with recombinant plasmid pET28a-LvCrustinIa-2, pET28a-LvCrustinIa-2-WAP, or pET32a; lane 2, total protein of *E. coli* with recombinant plasmid pET28a-LvCrustinIa-2, pET28a-LvCrustinIa-2-WAP, or pET32a after IPTG induction; lane 3, purified recombinant protein.

The recombinant plasmid pET28a-LvCrustinIa-2 and pET28a-LvCrustinIa-2-WAP were expressed in *E. coli* BL21 (DE3). After IPTG induction, SDS-PAGE analysis of the whole cell lysates revealed that both rLvCrustinIa-2 and rLvCrustinIa-2-WAP were mainly expressed as supernatant proteins (Figure 3). The purified rLvCrustinIa-2 and rLvCrustinIa-2-WAP exhibited a distinct band at approximately 18 kDa and 11 kDa, respectively, which was consistent with the predicted molecular weight of fusion proteins. The transformant with pET-32a was induced, and its recombinant thioredoxin (rTrx) was also successfully expressed with the molecular mass of 22 kDa. The purified rLvCrustinIa-2, rLvCrustinIa-2-WAP, and rTrx proteins were at the concentrations of 1.86 mg mL^−1^, 1.61 mg mL^−1^, and 1.81 mg mL^−1^, respectively.

### 3.4. Antimicrobial Activity of LvCrustinIa-2 Depending on the WAP Domain

The antimicrobial activity of rLvCrustinIa-2, rLvCrustinIa-2-WAP, and synthetic LvCrustinIa-2-CR against several bacteria and a fungus was evaluated using the minimum inhibitory concentration (MIC) assay (Table 2). The purified rLvCrustinIa-2 exhibited inhibitory effects against the tested Gram-positive bacteria, *S. aureus* S7, *S. delphini* Sd2, and *M. luteus* M2, and two Gram-negative bacteria, *V. parahaemolyticus* Pa6 and *V. alginolyticus* L59, but had no effect on the fungus *P. pastoris* GS115 or two Gram-negative bacteria, *P. aeruginosa* P25 and *E. tarda* E3. A stronger antimicrobial activity of rLvCrustinIa-2 was detected against Gram-positive bacteria with MIC values of 10.7 μM. Compared with rLvCrustinIa-2, rLvCrustinIa-2-WAP showed weaker antibacterial activity against *S. delphini* Sd2 and *M. Luteus* M2 with MIC values of 15.4 μM, but had higher antibacterial activity against *V. parahaemolyticus* Pa6, *V. alginolyticus* L59, and *S. aureus* S7 with MIC values of 7.7 μM. No obvious antimicrobial activity of synthetic LvCrustinIa-2-CR and rTrx was observed.

### 3.5. Microbial Binding Activity of LvCrustinIa-2

**Figure 4:**
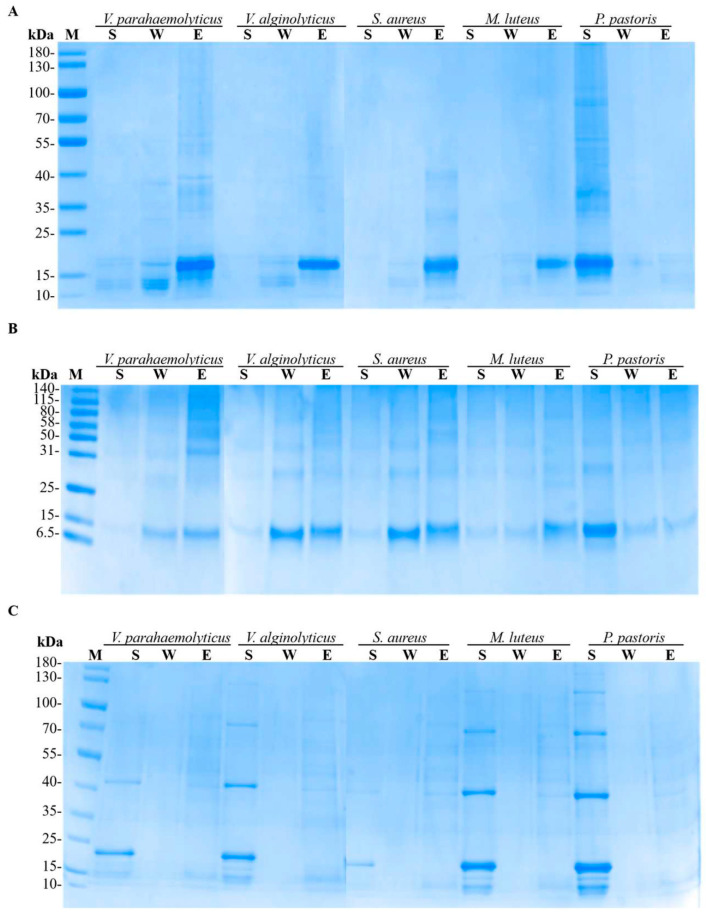
Binding activities of rLvCrustinIa-2 (**A**), rLvCrustinIa-2-WAP (**B**), and rTrx (**C**) to microorganisms. The supernatants (S), washed (W), and eluted (E) fractions were examined by SDS-PAGE.

The binding activity of rLvCrustinIa-2, rLvCrustinIa-2-WAP, and rTrx to various microorganisms was analyzed by SDS-PAGE (Figure 4). For rLvCrustinIa-2, most protein was detected in the eluted fractions of the test bacteria with only slight bands seen in the washed fractions (Figure 4A). As for rLvCrustinIa-2-WAP, distinct bands were observed in both the eluted and washed fractions of bacteria, along with minimal detection in the supernatants (Figure 4B). After incubation with fungus *P. pastoris*, both rLvCrustinIa-2 and rLvCrustinIa-2-WAP protein showed clear bands in the supernatant. The results demonstrated that both rLvCrustinIa-2 and rLvCrustinIa-2-WAP could bind to Gram-negative bacteria *V. parahaemolyticus* and *V. alginolyticus*, and Gram-positive bacteria *S. aureus* and *M. luteus*, but not to fungus *P. pastoris*. Notably, rLvCrustinIa-2 exhibited stronger microbial binding activity than rLvCrustinIa-2-WAP. In contrast, the negative control rTrx showed no binding affinity for the tested microorganisms due to the clear bands only in the supernatant fractions (Figure 4C).

### 3.6. Effects of LvCrustinIa-2 on Bacterial Morphology and Membrane Integrity

**Figure 5:**
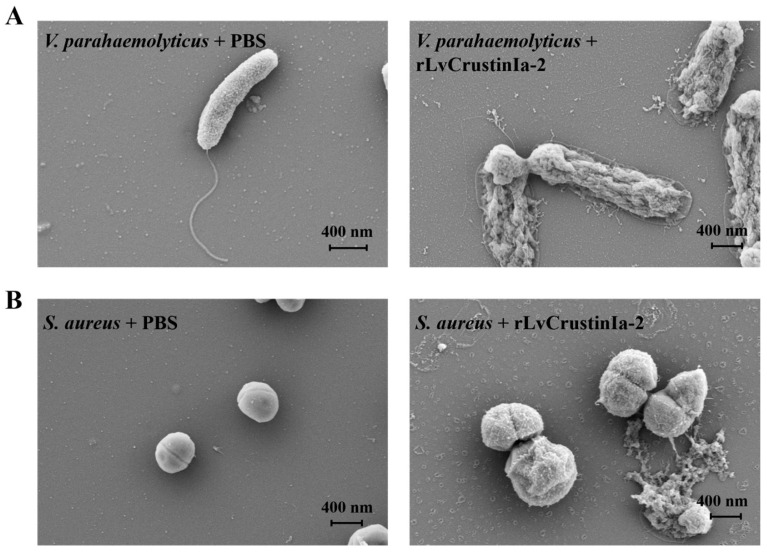
Morphological changes in bacterial cells treated with rLvCrustinIa-2. *V. parahaemolyticus* (**A**) and *S. aureus* (**B**) were incubated with rLvCrustinIa-2 or PBS. Bacterial morphology was examined by a scanning electron microscope.

Electron microscopy showed the treatment of *V. parahaemolyticus* or *S. aureus* with rLvCrustinIa-2-induced rapid changes in bacterial morphology. Compared to the control, the rLvCrustinIa-2-treated *V. parahaemolyticus* displayed severe structure destruction and release of cellular contents (Figure 5A). The rLvCrustinIa-2-treated *S. aureus* exhibited obvious swelling with wrinkled cell surfaces, and some bacterial cells underwent rupture (Figure 5B).

### 3.7. Chemotactic Activity of LvCrustinIa-2 on Shrimp Hemocytes

**Figure 6:**
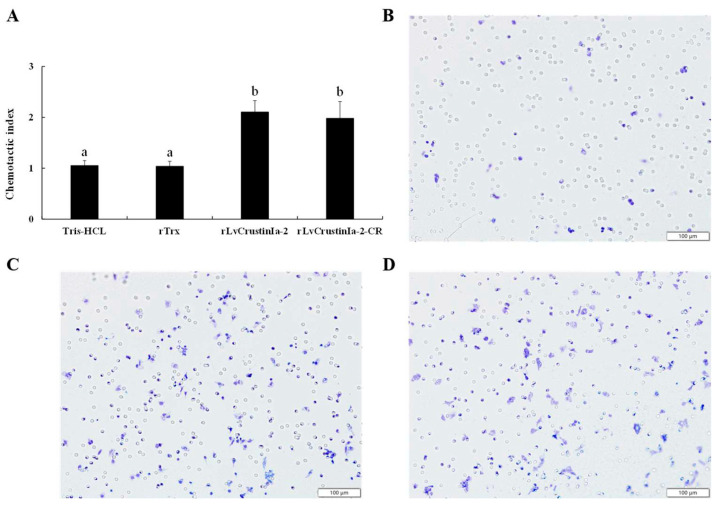
In vitro chemotactic activity of rLvCrustinIa-2 and synthesized LvCrustinIa-2-CR. (**A**) Chemotactic activity of rLvCrustinIa-2 and LvCrustinIa-2-CR to shrimp hemocytes. Tris-HCl buffer and rTrx treated groups were used as controls. Data are presented as mean ± S.D. from three independent experiments. Different lowercase letters identify treatment means that were significantly different from each other (*p* < 0.05). (**B**–**D**) Crystal violet staining solution staining shrimp hemocytes image (200×) after treated with rTrx, LvCrustinIa-2, and LvCrustinIa-2-CR. The scale bar is 100 µm.

To determine the chemotaxis of LvCrustinIa-2, we used Transwell assay system to evaluate chemotactic ability on shrimp hemocytes (Figure 6). Compared to the Tris-HCl buffer and rTrx control groups, rLvCrustinIa-2 at 0.15 mg mL^−1^ and LvCrustinIa-2-CR at 0.17 mg mL^−1^ could significantly accelerate the migration of hemocytes into the lower chamber (*p* < 0.05). rLvCrustinIa-2 with CI value of 2.1 exhibited higher chemotactic ability for shrimp hemocytes than LvCrustinIa-2-CR with CI value of 1.9. Meanwhile, rLvCrustinIa-2-WAP failed to show any chemotactic activity for shrimp hemocytes.

### 3.8. LvCrustinIa-2 Knockdown and Its Effect on the Expression of Immune Genes

**Figure 7:**
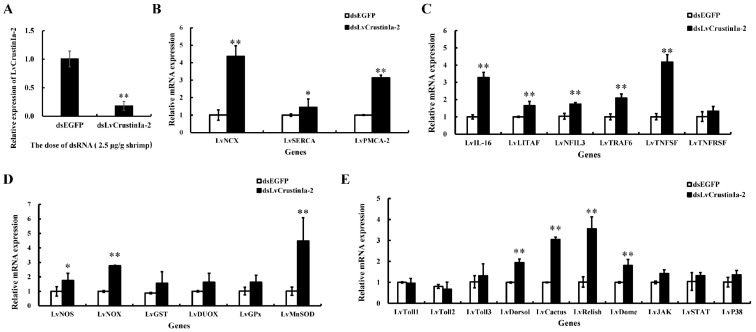
Expression pattern of *LvCrustinIa-2* and immune genes post-RNAi. (**A**) The knockdown efficiency of *LvCrustinIa-2* in shrimp hemocytes at 48 h post-dsRNA injection. The immune genes are involved in (**B**) calcium transport, (**C**) inflammation, (**D**) redox, and (**E**) NF-κB, JAK/STAT and MAPK signaling pathways. Data are shown as mean ± S.D. (n = 3). Asterisks indicate the significant differences compared with control at the same sampling point (* *p* < 0.05, ** *p* < 0.01).

*LvCrustinIa-2* was knocked down by dsRNA-mediated RNAi. Compared with dsEGFP group, the expression of *LvCrustinIa-2* in hemocytes was significantly downregulated in shrimps injected with *dsLvCrustinIa-2* at a dose of 2.5 µg/g shrimp. The knockdown efficiency of *LvCrustinIa-2* was 81.9% (Figure 7A).

After *LvCrustinIa-2* gene knockdown, the expression levels of calcium transport genes *LvNCX*, *LvSERCA,* and *LvPMCA-2*, inflammation-related genes *LvIL-16*, *LvLITAF*, *LvNFIL3*, *LvTRAF6,* and *LvTNFSF*, redox genes *LvNOS*, *LvNOX,* and *LvMnSOD*, NF-κB signaling pathway genes *LvDorsal*, *LvCactus,* and *LvRelish*, and JAK/STAT pathway genes *LvDome* were significantly upregulated compared to dsEGFP control group (Figure 7B–E). There were no significant effects on the expression levels of *LvTNFRSF*, *LvGST*, *LvDUOX*, *LvGPx*, *LvToll1-3*, *LvJAK*, *LvSTAT,* and *LvP38* after the knockdown of *LvCrustinIa-2*.

## 4. Discussion

Crustins are important immune effectors in crustaceans and constitute the first line of defense against pathogen infection [27]. In the present study, LvCrustinIa-2, like the type Ia crustins, contains an N-terminal signal peptide, a cysteine-rich region with four conserved cysteine residues, and a C-terminal WAP domain with eight conserved cysteine residues. Structural prediction analysis revealed that LvCrustinIa-2 exhibited cationic patchy surface and amphipathic structure, which indicated that LvCrustinIa-2 was a cationic and amphipathic protein. Consistent with the established mechanism of action of AMPs [40], these structural features of LvCrustinIa-2 might facilitate binding to and interaction with pathogen cell membranes, ultimately contributing to its antimicrobial activity.

*LvCrustinIa-2* was highly expressed in hemocytes and significantly upregulated in response to *V. parahaemolyticus* and WSSV challenge. These results indicated that *LvCrustinIa-2* could play an important role in hemocyte-mediated immune defense against pathogen infection. Similar expression profiles have been reported for crustins in other crustaceans, such as *MjCRS* in prawn *Marsupenaeus japonicus* [41], *MrCrs* in prawn *Macrobrachium rosenbergii* [42], *Plcrustin* and *Plcrustin2* in crayfish *Pacifastacus leniusculus* [43], and *CrusEs2* in mitten crab *Eriocheir sinensis* [44].

The antimicrobial spectrum of most reported type I crustins is restricted to Gram-positive bacteria [22,26,45,46,47]. However, rLvCrustinIa-2 showed stronger antimicrobial activities against Gram-positive bacteria in this study. This finding aligned with results from several crustins, such as CruHa1 and CruHa2 from spider crab *Hyas araneus* [48], CqCrs from crayfish *Cherax quadricarinatus* [49], and PtCrustin2 and PtCrustin3 from crab *Portunus trituberculatus* [50]. Consistently, rLvCrustinIa-2 showed strong binding affinity to both Gram-positive and Gram-negative bacteria. Collectively, these results demonstrated that LvCrustinIa-2 could act as an AMP capable of binding and eliminating invading pathogens in shrimp hemolymph.

The WAP domain of crustins has been identified as the key functional structure for their antimicrobial response and protease inhibition [27,51]. Previous studies have demonstrated that the bactericidal activity of crustins can be abolished if the WAP domain lacks integrity or its disulfide bonds were disrupted [52,53]. Consistent with these findings, our study revealed that recombinant WAP domain of LvCrustinIa-2 (rLvCrustinIa-2-WAP), but not the synthetic cysteine-rich region (LvCrustinIa-2-CR), displayed bactericidal activity, further confirming the essential role of the WAP domain in crustin function. Interestingly, the mature protein rLvCrustinIa-2 exhibited significantly stronger microbial binding activity than rLvCrustinIa-2-WAP, suggesting the cysteine-rich region might enhance binding between the protein and target bacteria.

To date, no crustacean AMPs with confirmed chemotactic activity have been reported. Our study demonstrated for the first time that rLvCrustinIa-2 significantly enhanced the chemotactic activity of hemocytes. Similar observations have been reported in mollusk AMPs, such as Rpdef1α from *Ruditapes philippinarum* [18], HdMac from *Haliotis discus hannai* [19], and VpMacin-1 and VpMacin-2 from *Venerupis philippinarum* [20]. Accumulating evidence suggested that AMPs and chemokines share a common origin due to their overlapping functions [54]. For example, human cationic antimicrobial peptide LL-37 could mediate chemotaxis for specific leukocyte subsets [55]. Human chemokines CXCL4 and CCL28 were found to act as antimicrobial peptides in addition to their chemoattractant role [56,57,58]. Notably, in our study, the chemotactic activity was specifically mediated by the cysteine-rich region (LvCrustinIa-2-CR), with no detectable activity observed in the WAP domain (LvCrustinIa-2-WAP). This indicates that the chemotactic activity of LvCrustinIa-2 was primarily mediated by the cysteine-rich region. The molecular mechanism underlying crustin-mediated chemotaxis, especially the roles of disulfide bonds, requires further investigation.

Calcium ion flux has been documented to play a crucial role in cell migration and chemotaxis [59,60,61]. To investigate the potential involvement of *LvCrustinIa-2* in calcium signaling, we detected the expression of key calcium transport genes in *LvCrustinIa-2*-knockdown shrimp. Our results showed that the knockdown of *LvCrustinIa-2* could upregulate the expression of three major calcium transporters *LvNCX*, *LvSERCA,* and *LvPMCA-2*, implying that *LvCrustinIa-2* might influence the calcium ion flux gradients during chemotaxis. Additionally, some fish AMPs, such as GRN-4 from Mozambique tilapia [62] and epinecidin-1 from *Epinephelus coioides* [63], have been reported to be involved in the regulation of inflammatory responses [64]. A recent study in deep-sea shrimp showed Crus2 could impair the ability of LPS and LTA to induce the release of IL-6, IL-1β and TNF-α from murine J774.1 cells [65]. In the present study, most NF-κB pathway genes, inflammation genes, and redox-related genes were obviously upregulated in the *LvCrustinIa-2*-knockdown shrimp. These results indicate that LvCrustinIa-2 might participate in the inflammatory regulation of shrimp.

## 5. Conclusions

In conclusion, LvCrustinIa-2, a cationic and amphipathic AMP, was upregulated after challenge with *V. parahaemolyticus* and WSSV. The recombinant LvCrustinIa-2 displayed stronger antimicrobial activity against Gram-positive bacteria, and demonstrated significant chemotactic activity towards shrimp hemocytes in vitro. Specifically, the recombinant WAP domain primarily mediated bactericidal activity and bacterial surface binding, whereas the synthetic cysteine-rich region was associated with chemotactic activity. Moreover, the knockdown of *LvCrustinIa-2* resulted in upregulated expression of calcium transport regulators, inflammation-related genes, antioxidant enzymes, and NF-κB pathway genes. To our knowledge, this is the first report of a crustacean AMP with chemotactic activity. These findings not only reveal a novel function of crustacean crustins but also provide insights into the evolutionary relationship between AMPs and chemokines.

## Figures and Tables

**Table 1:** Primers used in this study.

Primers Name	Sequence (5′-3′)	PCR Objective
LvCrustinIa-2-F	AGACAACATGCAGGGTCTCG	Gene cloning
LvCrustinIa-2-R	CCCTTCTCAACCGAACTGG	Gene cloning
dsLvCrustinIa-2-F	TAATACGACTCACTATAGGGAGACAACATGCAGGGTCTCG	dsRNA
dsLvCrustinIa-2-R	TAATACGACTCACTATAGGGCCCTTCTCAACCGAACTGG	dsRNA
LvCrustinIa-2-qF	CCCACAAGTCCGTCCCACCT	Real-time PCR
LvCrustinIa-2-qR	CTCCCAGACACCTGTCGAAGCAG	Real-time PCR
rLvCrustinIa-2-F	AATGGGTCGCGGATCCAGCTTCCCCGGCGCCACAGCC	Recombinant expression
r LvCrustinIa-2-R	CTCGAGTGCGGCCGCAAGCTTACCGAACTGGTTGTAG	Recombinant expression
rLvCrustinIa-2-WAP-F	AATGGGTCGCGGATCCAAGCCCCTCGACTGCCCACAA	Recombinant expression
rLvCrustinIa-2-WAP-R	GTGCGGCCGCAAGCTTGGAAGGGGGCTTGCACACGT	Recombinant expression
18S-F	TATACGCTAGTGGAGCTGGAA	Real-time PCR
18S-R	GGGGAGGTAGTGACGAAAAAT	Real-time PCR
dsEGFP-F	TAATACGACTCACTATAGGGCAGTGCTTCAGCCGCTACCC	dsRNA
dsEGFP-R	TAATACGACTCACTATAGGGAGTTCACCTTGATGCCGTTCTT	dsRNA
T7 promoter	TAATACGACTCACTATAGGG	Sequencing
T7 terminator	GCTAGTTATTGCTCAGCGGT	Sequencing
M13-47	CGCCAGGGTTTTCCCAGTCACGAC	Sequencing
RV-M	GAGCGGATAACAATTTCACACAGG	Sequencing
LvNCX-qF	ATCGGTCTGAAGGACTCGG	Real-time PCR
LvNCX-R	TGGACATTGTGGTAGATAGCA	Real-time PCR
LvPMCA-2-qF	CGGAGGCTACCGCATTTAT	Real-time PCR
LvPMCA-2-qR	CACTTCAGGACGCACAGGA	Real-time PCR
LvSERCA-qF	CCGTATTGGTGTGTTTGGTG	Real-time PCR
LvSERCA-qR	TGGATTTGTGGAAAGGCTCG	Real-time PCR
LvLITAF-qF	GCAGTCAACGCACATGATCT	Real-time PCR
LvLITAF-qR	TTGTATTTGCCCAGGAAAGC	Real-time PCR
LvIL-16-qF	AGCAAGAGCCTCGTGTCAGAC	Real-time PCR
LvIL-16-qR	CCTCCAGAGAAAAGCCCAGT	Real-time PCR
LvNIFL3-qF	ATTATGGTTGCTGAGACGGTGA	Real-time PCR
LvNIFL3-qR	GATGTGGGGCGAGTAGTTGG	Real-time PCR
LvTRAF6-qF	ACATCACCAATCCCAGAG	Real-time PCR
LvTRAF6-qR	GTCAGCACCGCCTTTATC	Real-time PCR
LvTNFSF-qF	CAGAGCCGTCAAGAAGATCC	Real-time PCR
LvTNFSF-qR	TGAGGGAGTACTTCCGGTTG	Real-time PCR
LvTNFRSF-qF	AAAGAGGAACGTGGTCATGG	Real-time PCR
LvTNFRSF-qR	CACTCCTTTCCCCACTGTGT	Real-time PCR
LvMnSOD-qF	ATTGCCGCTACGAAGAAG	Real-time PCR
LvMnSOD-qR	AGATGGTGTGGTTCAAGTG	Real-time PCR
LvGPx-qF	GCACCAGGAGAACACTAC	Real-time PCR
LvGPx-qR	TTCCAGGCAATGTCAGAG	Real-time PCR
LvGST-qF	AGAAAAACTACCCTGTCGG	Real-time PCR
LvGST-qR	CCTTGCTCTGCGTTATCTT	Real-time PCR
LvDUOX-qF	GACTTGGCAGCAAACCTA	Real-time PCR
LvDUOX-qR	TGCGGGAAAGGTCGTAGAT	Real-time PCR
LvNOX-qF	CCAACGATGTGCCTGATAGTG	Real-time PCR
LvNOX-qR	ATGTCGGTCTTCTGAAGGGCT	Real-time PCR
LvNOS-qF	GAGCAAGTTATTCGGCAAGGC	Real-time PCR
LvNOS-qR	TCTCTCCCAGTTTCTTGGCGT	Real-time PCR
LvToll1-qF	CTATTGTGGTGCTTTCGT	Real-time PCR
LvToll1-qR	TGGAGATGTACAGTCGTAAC	Real-time PCR
LvToll2-qF	CATGCCTGCAGGACTGTTTA	Real-time PCR
LvToll2-qR	GGCCTGAGGGTAAGGTCTTC	Real-time PCR
LvToll3-qF	GTGAATCTGACCCGAGTTGA	Real-time PCR
LvToll3-qR	TGCTGCCTTCGGTGTTCTA	Real-time PCR
LvCactus-qF	GCCTGTCTTACGCCCCT	Real-time PCR
LvCactus-qR	CCGTCCGACCACTCTTG	Real-time PCR
LvRelish-qF	CATGCAAGACTTCGCAA	Real-time PCR
LvRelish-qR	CTGGTAATGTAACAGGACG	Real-time PCR
LvDorsal-qF	TGGGGAAGGAAGGATGC	Real-time PCR
LvDorsal-qR	CGTAACTTGAGGGCATCTTC	Real-time PCR
LvDOME-qF	CTCAGGCTATGTTTCTCAGGATTCA	Real-time PCR
LvDOME-qR	CACGGCAGTTCCTTTATGGTCT	Real-time PCR
LvJAK-qF	CCTTAATTCGAGCGCAATGGG	Real-time PCR
LvJAK-qR	CTAGCGACAGAGGGTTTAGCG	Real-time PCR
LvSTAT-qF	TATATCCGAATGTGCCTAAG	Real-time PCR
LvSTAT-qR	ATAGTTTGTGGTGTGTTGGG	Real-time PCR
Lvp38-qF	GTCGGCTCGCAACTACATAC	Real-time PCR
Lvp38-qR	CCGTTACACGCCTTTCACT	Real-time PCR

**Table 2:** Antimicrobial activities and minimal inhibitory concentrations (MICs) of rLvCrustinIa-2 and rLvCrustinIa-2-WAP.

Microorganism	MIC (µM)	
	rLvCrustinIa-2	rLvCrustinIa-2-WAP
Gram-negative bacteria		
*Vibrio parahaemolyticus* Pa6	21.4	7.7
*Vibrio alginolyticus* L59	21.4	7.7
*Pseudomonas aeruginosa* P25	>21.4	>30.7
*Edwardsiella tarda* E3	>21.4	>30.7
Gram-positive bacteria		
*Staphylococcus aureus* S7	10.7	7.7
*Staphylococcus delphini* Sd2	10.7	15.4
*Micrococcus luteus* M2	10.7	15.4
Fungus		
*Pichia pastoris* GS115	>21.4	>30.7

## Data Availability

The data presented in this study are available in the article.

## Supplementary Materials

The following supporting information can be downloaded at https://www.mdpi.com/article/10.3390/biom15071015/s1, Figure S1: Nucleotide and deduced amino acid sequences of LvCrustinIa-2 from *Litopenaeus vannamei*. The start codon (ATG) and stop codon (TGA) are marked with red line and asterisk (*), respectively. The signal peptide is indicated with red box. The WAP domain is shaded with gray. The conserved cysteine residues are marked with black boxes.
