# Supplementary figures and images for: A Type Ia Crustin from the Pacific White Shrimp Litopenaeus vannamei Exhibits Antimicrobial and Chemotactic Activities
Source: Biomolecules. 2025 Jul 14;15(7):1015. doi: 10.3390/biom15071015 (PMC12292379; doi:10.3390/biom15071015)

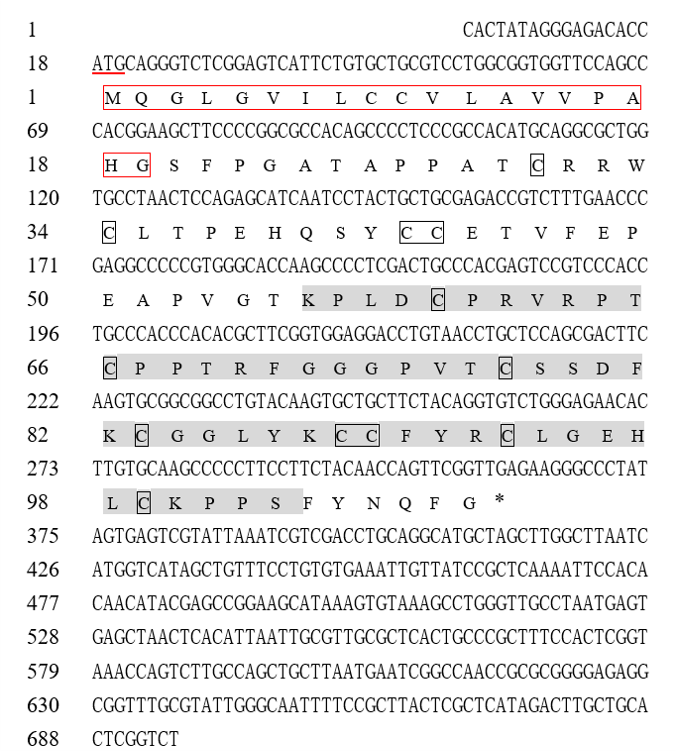

Supplement: Supplementary file 1 [file biomolecules-15-01015-s001.zip › biomolecules-3657455-supplementary.tif]
